# From Oxidised LDL to Potential Novel Applications in Gingival Crevicular Fluid Analysis

**DOI:** 10.3390/ijms262411924

**Published:** 2025-12-10

**Authors:** Matsuo Yamamoto, Takayuki Ootani, Hiroko Imai, Hiroyuki Itabe

**Affiliations:** 1 Department of Periodontology, Showa Medical University Graduate School of Dentistry, 2-1-1 Kitasenzoku, Ohta-ku, Tokyo 145-8515, Japan; t.otani@dent.showa-u.ac.jp; 2 Dentistry and Oral Surgery, Showa Medical University Karasuyama Hospital, 6-11-11 Kitakarasuyama, Setagaya-ku, Tokyo 157-8577, Japan; imai-hiroko2015@dent.showa-u.ac.jp; 3 Department of Biological Chemistry, Showa Medical University Graduate School of Pharmacy, 1-5-8 Hatanodai, Shinagawa-ku, Tokyo 142-8555, Japan; h-itabe@pharm.showa-u.ac.jp

**Keywords:** periodontitis, oxidative stress, GCF, oxidised LDL, apoB

## Abstract

Gingival crevicular fluid (GCF) reflects both local periodontal inflammation and systemic conditions. This review highlights the role of oxidative stress, oxidised low-density lipoprotein (oxLDL), and apolipoprotein B (apoB) as molecular links between periodontitis and metabolic disorders. Elevated GCF levels of oxLDL and apoB indicate enhanced vascular permeability and local oxidative modification, particularly in diabetes. Furthermore, oxLDL promotes the formation of neutrophil extracellular trap (NET) via connecting oxidative stress with immune-mediated tissue injury. These insights establish GCF as a valuable, non-invasive biomarker for understanding the interplay between periodontal and systemic diseases.

## 1. The History of Periodontal Pocket Probing and Gingival Crevicular Fluid (GCF)

Periodontal disease is an inflammatory disease caused by infection with periodontal pathogens, but it is known to affect not only local infection but also the overall interaction between periodontal disease and systemic diseases. Gingival crevicular fluid (GCF) has been established as a valuable non-invasive biomarker for understanding the interaction between periodontal disease and systemic diseases. However, this review focuses on GCF analysis to provide greater transparency regarding how the relevant evidence was selected and integrated into this review; thus, we have summarised the literature search strategy below.

The literature for this review was identified through searches of PubMed and Web of Science using combinations of keywords related to “oxidised LDL,” “gingival crevicular fluid,” “periodontitis,” “NETosis,” and “neutrophil function.” Publications from 1977 to 2024 were considered to reflect both historical background and contemporary advances. Studies were included if they reported original data or provided mechanistic insights relevant to oxidative stress, lipid oxidation, or innate immune responses in periodontal or systemic conditions. Clinical case reports, non-peer-reviewed sources, and papers unrelated to lipid oxidation or innate immunity were excluded. This narrative review did not apply strict systematic review criteria but aimed to integrate representative, mechanistically informative evidence across the available literature.

The gingival crevicular space constitutes a boundary between the external environment and the internal body. Numerous microorganisms exist within the gingival crevicular space, forming a bacterial flora. The junctional epithelium functions as a barrier for biological defence because plasma components extrude from the dentogingival plexus in the connective tissue adjacent to the junctional epithelium and permeate the junctional epithelium. These plasma components supply oxygen and nutrients to the junctional epithelial tissue. The metabolic products and components of damaged cells are removed in the fluid from the junctional epithelium. This tissue fluid passes through the junctional epithelium and is discharged into the gingival sulcus, where the fluid mixes with saliva. This fluid is termed GCF. GCF contains antimicrobial substances such as β-defensins and LL-37, antibodies, and polymorphonuclear leukocytes (PMNs). These components strongly contribute to the innate immune function of the junctional epithelium [1].

The gingival crevicle is called a periodontal pocket when periodontal tissues deepen due to inflammation caused by microbial invasion. This environment experiences under-goes large biological changes due to infections and inflammation. The damage to perio-dontal tissues is assessed as the loss of the fibrous attachment that anchors the tooth to the alveolar bone. Attachment loss is measured as the distance from the cementoenamel junc-tion to the bottom of the pocket. Several indicators are available for assessing attachment loss in clinical practice and are referred to as clinical parameters. These parameters in-clude pocket probing depth, bleeding on probing, clinical attachment level, tooth mobility, and plaque control records. Periodontal tissues and radiological images re used to objec-tively evaluate the extent of tissue destruction. However, these indicators only provide a cross-sectional view of the condition of the damaged tissue. Additional microbiological or biochemical measurements must be considered to obtain the information required for pre-dicting disease activity, healing, and prognosis at the sites affected by periodontal disease.

**Figure 1:**
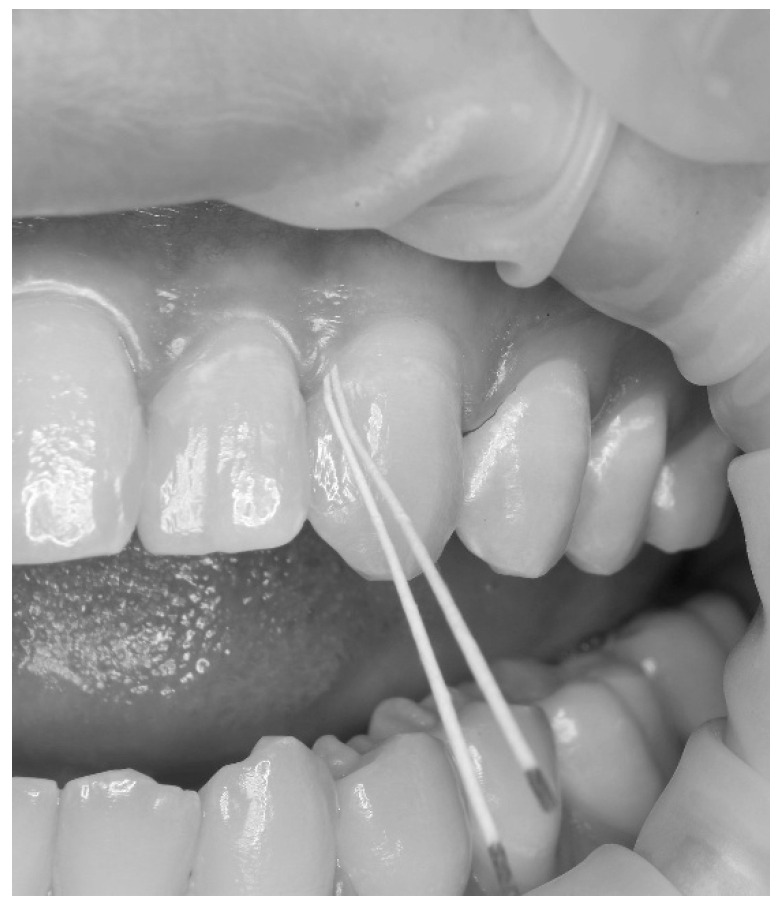
GCF samples collected using sterile paper points.

The quantity of GCF increases with altered vascular permeability, paralleling the progression of gingivitis. Consequently, GCF has been studied as an indicator of inflammation. The Periotron device (Oraflow Inc) was developed as a measuring instrument, significantly contributing to the chairside assessment of periodontal status [2,3]. Subsequently, attention shifted to analysing the composition of GCF to understand the conditions within the formed periodontal pocket. GCF can be collected by inserting clean paper points or absorbent strips into the gingival sulcus, utilising capillary action (Figure 1). GCF primarily consists of plasma components extravasated from the dentogingival plexus, alongside metabolic products from periodontal tissue cells and antimicrobial peptides. Consequently, GCF reflects the local tissue state while also being strongly correlated with the condition of plasma circulating throughout the body. GCF contains not only microbial cells and their constituents but also numerous host-derived components such as antibodies, cytokines, and cellular constituents. These serve as biomarkers useful for understanding disease mechanisms and monitoring host responses. According to Buduneli M et al., the components present in GCF that serve as biomarkers for the diagnosis of periodontal disease can be classified into four main categories [4]:

1. Inflammatory mediators;
2. Host-derived enzymes;
3. Bone metabolism markers;
4. Oxidative stress markers.

When periodontal tissues become inflamed, subsequent destruction of soft and hard tissues progresses. Therefore, biochemical analysis of GCF enables a more detailed understanding of the pathological state. Representative examples for measurement include inflammatory cytokines such as IL-1β and TNF-α, and chemokines such as IL-8. Additionally, host-derived enzymes such as matrix metalloproteinases (MMPs) and their inhibitors, TIMP, along with components indicative of cell destruction like aspartate aminotransferase (AST) and alkaline phosphatase (ALP), are also measured. Representative bone metabolism markers include Receptor Activator of Nuclear Factor-κB ligand (RANKL), osteoprotegerin (OPG), osteocalcin, N-telopeptide, C-telopeptide, and deoxypyridinoline. Comprehensive evaluation of these markers has enabled numerous studies to conduct detailed pathological analyses. In recent years, miRNA analysis has also advanced, enabling more detailed analysis of tissue function. Polymorphonuclear leukocytes (PMNs) migrate into the periodontal tissues adjacent to the gingival sulcus or periodontal pocket to eliminate invading microorganisms, generating substantial amounts of reactive oxygen species (ROS). While ROS contributes to the destruction and elimination of invading foreign substances, excessive exposure to surrounding cells causes severe tissue damage. PMNs collected from patients with rapidly progressive periodontitis demonstrated increased ROS production in response to opsonised bacteria compared to healthy individuals [5]. Thus, oxidative stress must be evaluated for identifying the mechanisms underlying inflammation-induced tissue destruction. To highlight the distinctive diagnostic value of GCF, we briefly compared it with serum and saliva. Whereas serum reflects systemic conditions and saliva enables convenient non-invasive sampling, GCF uniquely provides site-specific biochemical information from periodontal tissues. At the same time, GCF collection is inherently subject to volume variability and potential contamination with saliva or blood, which should be considered when interpreting biochemical measurements. This comparison is summarised in Table 1.

## 2. Periodontal Disease and Oxidative Stress

ROS are physiologically generated during cellular respiration in normal tissues. Furthermore, to maintain tissue homeostasis, a constitutive low-grade inflammatory response is present in clinically healthy tissues at levels tolerated by the host. However, when bacterial infection induces tissue inflammation, cells in an inflamed state markedly increase the production of cytokines, proteolytic enzymes, and ROS. One of the primary phenomena of innate immunity is the infiltration of PMNs. These leukocytes are crucial for suppressing periodontal pathogens [6]. In particular, neutrophils, which constitute the majority of PMNs within the periodontal pocket, are responsible for the elimination of bacteria and their products through diverse functions, including phagocytosis, degranulation, and the release of myeloperoxidase and ROS into the extracellular space. Furthermore, intracellular ROS activate IL-1β and IL-18 [7]. Various inflammatory mediators—such as cytokines and chemotactic factors—are also secreted by gingival epithelial cells and by fibroblasts within the gingival connective tissue. Such responses are essential for eliminating locally invading bacteria and their products and for resolving bacterial infections.

Periodontal disease progresses chronically due to persistent infection by oral plaque bacteria. Consequently, the host inflammatory response varies. Inflamed periodontal tissues are continuously exposed to ROS, and this sustained inflammation further contributes to host-tissue damage [7]. Furthermore, persistent intracellular ROS damage cellular proteins, DNA, and lipids, leading to progressive cell and tissue damage. In the epithelium—the first line of defence—intercellular adhesion becomes disrupted, and membrane permeability increases. Consequently, epithelial barrier function becomes impaired [7]. As a result, the biological defence mechanisms of the oral epithelium become compromised, which contributes to the pathogenesis of periodontal disease [8]. In addition, systemic metabolic disturbances—such as diabetes, obesity, dyslipidemia, and ageing—may further elevate the oxidative burden by impairing antioxidant capacity and promoting chronic low-grade inflammation [9]. Dysbiotic subgingival biofilms dominated by anaerobic pathogens—particularly *Porphyromonas gingivalis*, a keystone species that promotes immune subversion and sustained inflammation—contribute to self-perpetuating oxidative signalling and reduced host redox defence [10]. Moreover, lifestyle and behavioural factors—including smoking, unhealthy diets, inadequate oral hygiene, psychological stress, and excessive alcohol intake—have been linked to increased oxidative injury and reduced antioxidant capacity, collectively exacerbating oxidative stress within periodontal lesions [11].

In tissues exposed to excessive ROS generated by PMNs accumulating within inflamed sites, an oxidative-stress environment is established. Total antioxidant capacity (TAOC) within the periodontal pocket decreases as a result of periodontal inflammation. However, TAOC has been reported to recover following non-surgical periodontal treatment [12]. Because ROS are short-lived, tissue oxidative stress is assessed by measuring stable oxidatively modified products. ROS attack polyunsaturated fatty acids (PUFAs), essential structural components of cell membranes, thereby inducing lipid peroxidation. This oxidative process generates malondialdehyde (MDA). MDA is a stable end product and is therefore readily measurable. Consequently, MDA has been widely adopted in numerous studies as an indicator of ROS-induced tissue damage. In periodontal disease, numerous studies have demonstrated an association between MDA—a representative marker of lipid peroxidation in GCF—and the severity of periodontitis [13,14]. However, the measurement of MDA is not highly sensitive as an indicator of oxidative stress. Furthermore, its measurement is technically challenging in GCF, which is obtainable only in minute quantities.

## 3. Association Between Metabolic Syndrome and Periodontal Disease

The Framingham Study in the United States reported that elevated blood levels of LDL-cholesterol (LDL-C) are strongly associated with the onset of cardiovascular disease (CVD) [15]. Metabolic conditions such as insulin resistance, hyperglycaemia, dyslipidaemia, and hypertension do not act independently but instead influence one another. The underlying mechanism has been reported to involve systemic insulin resistance and hyperinsulinemia [16,17]. Additional mechanisms involving adipokine secretion from adipose tissue, macrophage accumulation, and hyperglycaemia-induced vascular endothelial dysfunction have since been proposed. These findings have deepened our understanding that obesity, diabetes, and atherosclerosis are closely interrelated components of metabolic syndrome [18].

The mechanism can be summarised as follows. In obesity and dyslipidaemia, circulating levels of free fatty acids and LDL increase. Within adipose tissue, hypertrophied adipocytes experience hypoxia and increased oxidative stress. Consequently, the production of inflammatory cytokines increases. Macrophages accumulating in adipose tissue secrete inflammatory cytokines such as TNF-α, IL-6, and leptin. Conversely, the secretion of the anti-inflammatory adipokine adiponectin decreases. As a result, vascular endothelial function in the vessel wall becomes impaired. LDL particles deposit within the vessel wall, and macrophages infiltrating the intima accumulate lipids. This leads to the formation of foam cells and promotes vascular wall hardening. Furthermore, free fatty acids and inflammatory cytokines increase insulin resistance in tissue cells. Consequently, pancreatic β-cell function declines, leading to hyperglycaemia. In a hyperglycaemic state, vascular endothelial dysfunction, increased oxidative stress, enhanced glycation reactions, and heightened inflammatory activity occur. These processes accelerate atherosclerosis and further impair vascular endothelial function. Consequently, glucose uptake from the bloodstream decreases, making the hyperglycaemic state more likely to persist. Thus, obesity, diabetes, and atherosclerosis are closely interrelated.

Meanwhile, large-scale epidemiological studies have demonstrated an association between periodontal disease and lipid profiles. A positive correlation has been observed between the severity of attachment loss and Body Mass Index (BMI). Furthermore, patients with moderate periodontitis exhibit total cholesterol, LDL-C, and triglyceride levels that are approximately 8–39% higher than those of healthy controls. Total cholesterol and LDL-C levels have also been reported to correlate strongly with the Community Periodontal Index of Treatment Needs (CPITN). In periodontal disease, pathogens such as *Porphyromonas gingivalis* establish chronic infection within the periodontal pocket, leading to persistent local inflammation. This infection drives the release of pro-inflammatory cytokines, including IL-1β, IL-6, and TNF-α, into the bloodstream. These systemic mediators promote insulin resistance, adipose-tissue inflammation, and vascular endothelial dysfunction. Thus, metabolic syndrome and periodontitis are interrelated conditions linked through infection, inflammatory cytokines, and oxidative stress.

As described above, it is now well established that local periodontal inflammation is strongly correlated with systemic metabolic diseases. Moreover, an increasing number of studies have suggested that improving systemic metabolic conditions may, in turn, lead to improvements in the local periodontal environment.

Emerging host-modulatory approaches target oxidative stress at the gingival–crevicular interface. Nuclear factor erythroid 2–related factor 2 (Nrf2) -activating compounds such as sulforaphane—which is also available as an oral supplement—have been shown to reduce oxidative injury and preserve epithelial barrier integrity. In gingival epithelial cells, sulforaphane prevents H_2_O_2_-induced increases in epithelial permeability [19]. In addition, statins—owing to their pleiotropic anti-inflammatory and antioxidant actions—have demonstrated adjunctive benefits in non-surgical periodontal therapy, improving clinical outcomes and reducing oxidative markers [20]. Together, these findings suggest that Nrf2-based antioxidants and statin therapy help stabilise the epithelial barrier, suppress oxidative stress, and ultimately reduce oxLDL levels in both GCF and serum, thereby modulating the periodontal–systemic axis.

## 4. OxLDL and Periodontal Disease

**Figure 2:**
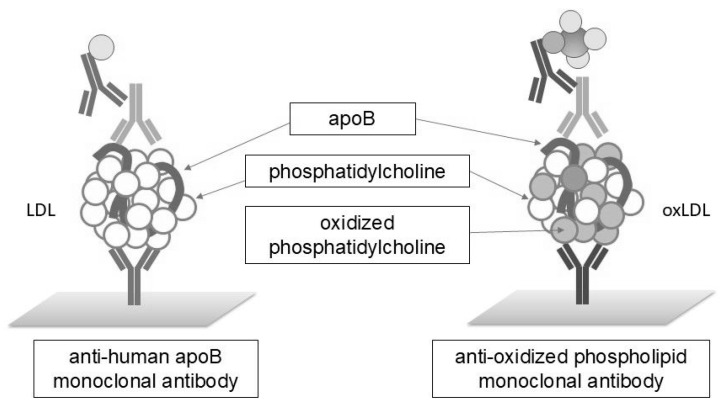
Principle of sandwich ELISA for detecting oxidised LDL. A monoclonal antibody against the oxidised phospholipid DLH3 specifically binds to oxidised phosphatidylcholine molecules. Diluted GCF samples were dispensed into microtiter wells precoated with the DLH3 and detected with an anti-ApoB polyclonal antibody and a horseradish peroxidase-conjugated secondary antibody.

Atherosclerosis is a prime example of a disease in which oxidative stress strongly influences its development and progression. OxLDL is one of the markers of this condition. GCF is a tissue fluid that seeps from the capillary network in the gingival marginal connective tissue into the gingival sulcus. GCF reflects not only the local periodontal environment but also plasma components. Therefore, LDL cholesterol was selected as an indicator of oxidative modification in GCF. LDL particles consist of a core of apoB protein and various lipid molecules, including cholesterol, triacylglycerol, and phosphatidylcholine. The PUFAs present in phosphatidylcholine serve as major substrates for oxidative modification. We developed a sandwich ELISA using the anti-oxidised phospholipid monoclonal antibody DLH3 and the sheep anti-human apoB polyclonal antibody. This method was employed to measure oxidised LDL in GCF (Figure 2).

GCF and blood were collected from patients with periodontitis who had no systemic diseases. The degree of oxidative stress was assessed by measuring oxLDL using oxPC and LDL using apoB, respectively, via a sandwich ELISA. The results showed that oxLDL was present in GCF, with its concentration approximately 17 times higher than that in plasma [21]. This finding is thought to reflect oxidative modification resulting from localised oxidative stress in the periodontal region. Next, we examined the effect of non-surgical periodontal treatment on the resolution of inflammation by assessing oxLDL levels in systemically healthy patients with chronic periodontitis (n = 11). OxLDL levels in GCF per tooth were approximately seven times higher at periodontitis sites (≥4 mm) compared to healthy sites (≤3 mm). In contrast, GCF volume at periodontitis sites was approximately twice that at healthy sites. GCF samples were collected on weeks 4 and 8 post-SRP and compared with baseline samples. Following treatment, oxLDL levels in GCF per tooth decreased to levels comparable with those at healthy sites. Concurrently, GCF volume decreased by approximately 55%, while ApoB and oxLDL levels decreased by 14.7% and 15.3%, respectively [22]. The reduction in detectable levels of ApoB and oxLDL (ng/site) exceeded the decrease in GCF volume, suggesting that, in addition to improved oxidative stress, protein leakage from tissues into GCF may also have been reduced. Numerous studies have reported similar decreases in oxidatively modified substances in GCF following reductions in periodontal inflammation.

The oxLDL levels in the GCF (oxLDL/LDL ratio) are markedly higher than in plasma, even in healthy individuals. This suggests that oxLDL increases in GCF via certain mechanisms. One possibility is that LDL extravasated into the GCF is more susceptible to oxidation. Another possibility is that oxLDL more readily permeates the junctional epithelium more readily than LDL, making it more likely to appear in GCF. In addition, proteins in GCF are more concentrated than in plasma. Local inflammation in the periodontal region enhances oxidative modification. Therefore, the observed increase in oxidised substances within GCF in the periodontal pocket is consistent with these mechanisms. At present, it is not fully understood which of these possibilities is correct, or whether both are valid. In vitro experiments have shown that the addition of exogenous oxLDL to the human gingival epithelial cell line Ca9-22 increases the secretion of IL-8 and PGE2 via the NF-κB pathway [23]. In addition to these observations, our previous studies further clarify how oxLDL actively contributes to periodontal tissue inflammation at the molecular level. We demonstrated that oxLDL strongly stimulates Ca9-22 and primary gingival keratinocytes to produce IL-8, IL-1β, and PGE_2_ through NF-κB and MAPK activation, accompanied by COX-2 and mPGES-1 upregulation and modulation of scavenger receptor expression [23,24]. These findings indicate that oxLDL functions not only as an oxidatively modified lipid but also as a direct pro-inflammatory mediator capable of amplifying epithelial cytokine and eicosanoid responses within periodontal pockets. Such epithelial activation promotes neutrophil recruitment and enhances local oxidative stress, reinforcing a positive feedback loop that further increases oxLDL generation.

Beyond epithelial cells, additional downstream mechanisms may link oxLDL to periodontal tissue destruction. First, oxLDL activates macrophages via scavenger receptors such as CD36 and SR-A, leading to the release of TNF-α, IL-1β, and matrix-degrading enzymes that contribute to connective-tissue breakdown [25]. Second, oxLDL induces endothelial dysfunction by reducing nitric oxide bioavailability and upregulating adhesion molecules, thereby facilitating leukocyte transmigration and lipoprotein extravasation into inflamed periodontal tissues [26]. Third, oxLDL has been shown to alter the RANKL/OPG balance toward a pro-resorptive state, enhancing osteoclast differentiation and promoting alveolar bone resorption [27,28]. Taken together, these pathways suggest that oxLDL is a biologically active driver of epithelial activation, macrophage-mediated inflammation, vascular dysregulation, and osteoclastic bone loss—providing a mechanistic basis for its elevated levels and pathogenic significance in GCF. OxLDL acts both as an oxidatively modified molecule and as an inflammatory mediator that induces tissue inflammation. As LDL oxidation progresses, oxLDL-mediated inflammatory responses may further intensify. This suggests the existence of a positive feedback loop.

We also investigated the impact of systemic disease on local oxidative stress in the periodontal region. The subjects comprised patients with diabetes (inpatient education group) with long-standing poor glycaemic control and healthy non-diabetic controls. Plasma and GCF were collected from shallow periodontal pockets (≤3 mm) for analysis (non-diabetic, n = 14; diabetic, n = 14). Periodontal disease was more severe in diabetic patients, and a correlation was observed between periodontal scores and diabetes-related parameters. GCF analysis was performed using the aforementioned sandwich ELISA method. Both the volume of collected GCF (GCF volume) and the protein concentration in GCF (GCF protein) showed a positive correlation with glycaemic parameters. In contrast, GCF volume and GCF protein did not correlate with lipidemic parameters, such as serum triglycerides (TG) or LDL-C. However GCF apoB (GCF-apoB) levels strongly correlateions with both glycaemic- and lipid-related parameters. Notably, the concentrations of apoB (GCF-apoB) and oxidised LDL (GCF-oxLDL) in GCF did not correlate with plasma apoB or oxidised LDL [29].

In plasma from diabetic patients, the concentration of MDA-LDL, an indicator of oxidative stress, did not substantially differ from that in healthy controls. In contrast, GCF volume was approximately 1.4-fold higher, GCF protein concentration was approximately 1.8-fold higher, and oxLDL concentration in GCF was approximately 2-fold higher. These findings suggest an increased leakage of oxLDL and proteins into GCF. Elevated plasma oxLDL may partly contribute to this increase, but locally specific mechanisms may also play a role. Furthermore, GCF apoB in samples from diabetic patients was approximately six times higher than in healthy controls. Considering that GCF protein concentration was only 1.8-fold higher, this may reflect not only local tissue inflammation but also alterations in the function of surrounding vascular endothelial cells. These results suggest that vascular permeability in the microcirculation of the junctional epithelium and the gingival epithelium is affected in diabetic patients. The permeability of plasma proteins and plasma lipoproteins is thought to be increased. However, the effects of vascular permeability on oxidative stress are limited. A mechanism that may be instructive in understanding this is receptor-dependent transcytosis of LDL in vascular endothelial cells. The macromolecular LDL may be selectively transported and involved in pathophysiology formation [30,31]. To summarise, oxidatively modified substances are present in GCF, and the degree of oxidative stress in GCF is greater than that in plasma. This is likely due to infection- and inflammation-related effects in the local periodontal environment. When inflammation subsides following basic periodontal treatment, both GCF volume and GCF protein decrease to levels comparable with those in healthy controls; however, the GCF-apoB and GCF-oxLDL levels became lower than those in healthy con-trols after this treatment.

**Figure 3:**
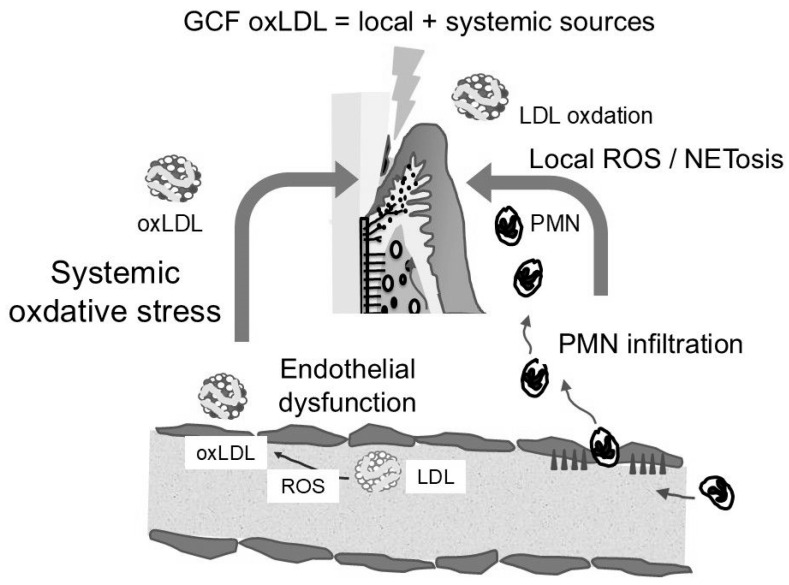
Interaction between local periodontal oxidative stress and systemic metabolic dysfunction, jointly contributing to elevated oxLDL levels in gingival crevicular fluid (GCF).

The evidence is increasingly supporting a bidirectional relationship between system-ic oxidative stress and periodontal inflammation. Circulating oxLDL may impair periodontal microvascular endothelial function, increasing permeability and promoting lipoprotein entry into GCF [32]. Conversely, periodontal inflammation can elevate systemic oxidative stress via cytokines and oxidatively modified proteins released into the bloodstream [33]. The markedly elevated oxLDL/LDL ratio in GCF—even at clinically healthy sites—indicates strong local oxidative modification [22]. However, comparison of GCF from shallow pockets in diabetic and healthy individuals revealed higher levels of GCF-apoB and GCF-oxLDL in diabetic samples, despite the absence of advanced periodontal tissue destruction. GCF-apoB levels were approximately sixfold higher than those in healthy individuals. The substantial increases in GCF-apoB and GCF-oxLDL observed in diabetic individuals suggest additional systemic contributions driven by metabolic dysfunction and endothelial permeability changes [29,34]. Thus, oxLDL in GCF reflects both local inflammatory oxidation and systemic oxidative status. Figure 3 illustrates how oxLDL detected in GCF reflects a composite signal arising from both local periodontal inflammation and systemic metabolic dysregulation.

Recent studies have highlighted the need for methodological standardisation in GCF oxLDL quantification, including harmonisation of sampling protocols, assay platforms, and reference ranges. Establishing these technical benchmarks will be essential for comparing results across studies and for translating oxLDL and apoB measurements into clinical decision-making tools. In addition, longitudinal studies are required to clarify how changes in GCF oxLDL/apoB correspond to periodontal disease progression, systemic metabolic conditions, and treatment outcomes.

Several limitations of oxLDL and apoB as biomarkers should be acknowledged. Their tissue specificity remains incomplete, as circulating oxLDL may originate from multiple systemic sources, and current assays are not yet optimised for cost-effective, routine use. Sensitivity may also be limited in detecting subtle inflammatory shifts, particularly in early-stage disease. Addressing these limitations—through assay standardisation, population-based validation, and development of more sensitive detection platforms—will be indispensable for establishing oxLDL and apoB as reliable biomarkers in both periodontal and systemic contexts.

**Figure 4:**
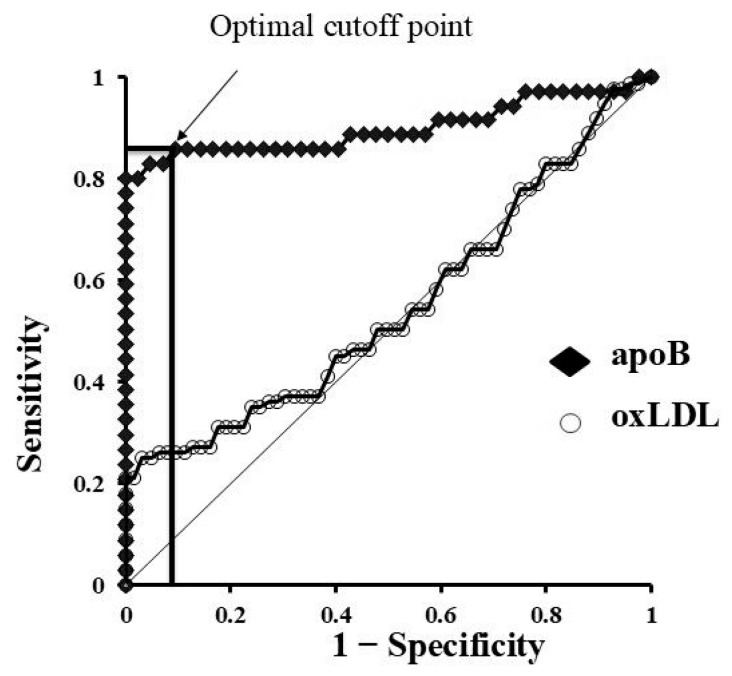
Diagnostic utility of apoB in gingival crevicular fluid (GCF) based on ROC analysis. ROC curve for apoB in GCF. The arrow indicates the optimal cutoff point that provides the best balance between sensitivity and specificity (Youden’s index).

These findings indicate that GCF contains abundant lipids and proteins that have traversed from plasma through vascular endothelial cells, periodontal connective tissue, and epithelial layers. The degree of translocation into GCF appears to differ depending on the substance. In particular, permeability changes in apoB were markedly enhanced by hyperglycaemia rather than by inflammation associated with periodontal disease (Figure 4). This suggests that GCF analysis may serve not only as a tool for periodontal research but also as a non-invasive diagnostic approach for systemic diseases.

## 5. Neutrophil Function and GCF

It has long been known that PMNs migrating into the gingival crevicular fluid remain viable and functionally active [35,36]. Approximately 75–80% of PMNs in GCF are viable and retain the ability to phagocytose [37]. Around 30% of PMNs in GCF undergo necrosis, whereas fewer than 1% undergo apoptosis [38]. However, recent advances in immunology have necessitated a revision of this interpretation. Neutrophils have been shown to release DNA and histones together with antimicrobial proteins (e.g., myeloperoxidase and elastase) in a web-like structure, thereby physically trapping and killing pathogens such as bacteria [39]. NETs have also been observed within periodontal pockets, where electron microscopy has confirmed the entrapment of bacteria [40]. The phenomenon known as NETosis appears to play a dual role, contributing both to the maintenance of periodontal tissue homeostasis and to tissue destruction in periodontitis [41]. Within periodontal pockets, NETosis is thought to be triggered by bacterial components [42], inflammatory cytokines [43], and ROS [44].

Our group previously demonstrated that oxLDL enhances NET formation by neutrophils [45]. Therefore, measuring oxLDL in GCF may not only serve as an indicator of oxidative tissue damage but also as a means of assessing the conditions promoting NETosis, which are intricately involved in both tissue defence and destruction.

The dual nature of NETosis is not limited to periodontal tissues but is also implicated in systemic inflammatory diseases. In rheumatoid arthritis (RA), NETosis-derived components—including extracellular DNA, histones, and myeloperoxidase (MPO)—together with lipid oxidation products such as oxidised LDL, synergistically amplify inflammatory responses and drive synovial membrane destruction. In systemic lupus erythematosus (SLE), excessive NETosis results in the release of NET-associated molecules (DNA, histones, MPO, and others) that serve as autoantigens, promoting autoantibody production and complement activation, thereby contributing centrally to disease pathogenesis [46]. Thus, when the mechanisms that regulate inflammation fail, immune reactions that ordinarily defend the host can become exaggerated and ultimately lead to damage and destruction of the host’s own tissues.

## 6. Potential Novel Applications in GCF Analysis

Recent findings from our group suggest that GCF may function not only as an indicator of local periodontal inflammation but also as a minimally invasive marker of systemic metabolic status [29]. While oxLDL in GCF reflects oxidative stress driven by periodontal inflammation, apoB in GCF exhibits a distinct biological pattern. In studies comparing systemically healthy individuals with patients with poorly controlled diabetes, GCF-apoB levels showed strong correlations with glycaemic and lipid-related parameters despite minimal periodontal destruction. The disproportionate sixfold elevation in apoB levels in individuals with diabetes—exceeding the modest rise in GCF protein—suggests selective enhancement of apoB-containing lipoprotein transport driven by diabetes-associated microvascular dysfunction [34]. Because GCF sampling is minimally invasive and chairside-feasible, apoB analysis may represent a novel oral fluid–based approach for evaluating systemic metabolic status.

GCF is likewise well-suited for next-generation diagnostic tools owing to its site specificity and reproducible sampling. Rapid analytical platforms such as microfluidic immunoassays could enable chairside quantification of oxLDL, apoB, or inflammatory mediators. Advances in electrochemical and optical biosensors may further enhance detection sensitivity using small GCF volumes, and future integration into intraoral wearable devices—such as smart mouthguards or sensor patches—could enable continuous monitoring of periodontal or metabolic fluctuations [47]. Combined saliva–GCF diagnostic approaches also hold promise, with saliva providing systemic information and GCF contributing site-specific biological signals. Such multi-layered analysis may improve early detection, risk assessment, and personalised periodontal management.

In addition, Omics-based profiling has expanded the molecular depth of knowledge of GCF. Proteomic, lipidomic, and metabolomic techniques can detect clusters of inflammatory, oxidative, and metabolic molecules that better characterise host response phenotypes than single-analyte measurements. Our proteomic investigation showed that GCF contains abundant neutrophil-derived proteins, inflammatory mediators, and oxidative stress–related molecules, reflecting active innate immune responses and tissue remodelling processes [48]. Machine-learning approaches can integrate these multidimensional datasets to identify optimal biomarker combinations, stratify patients by risk, or predict therapeutic outcomes [49]. Together, these strategies support the evolution of GCF from a single-marker research tool into a comprehensive molecular diagnostic platform for precision oral and systemic health.

## 7. Conclusions

GCF is a plasma-derived tissue fluid that is secreted into saliva; however, it is not merely a cleansing fluid for metabolic waste. Together with the junctional epithelium, GCF constitutes the first line of defence in periodontal tissues, functioning actively within the gingival sulcus and periodontal pockets. Analysis of GCF volume and composition enables the evaluation not only of the extent of tissue destruction but also of cellular function, its regulatory factors, and alterations in tissue physiology. In particular, the assessment of oxidative stress is crucial for understanding the mechanisms underlying NETosis. Therefore, GCF serves as an active biomarker that reflects diverse biological processes, highlighting the value of GCF as a tool for further biological investigations and enhancing predictive capacity and accuracy in clinical practice.

In addition to the established biological importance of GCF, the emerging technological and analytical innovations discussed in this review highlight the potential of using GCF as a minimally invasive tool for describing systemic metabolic conditions, positioning GCF as a promising component of future precision oral–systemic diagnostics.

## Figures and Tables

**Table 1:** Comparison of gingival crevicular fluid (GCF), serum, and saliva as biofluids for oxidative stress and inflammatory biomarker assessment.

Biofluid	Strengths	Limitations	Best Applications
Gingival crevicular fluid (GCF)	Site-specific; minimally invasive; reflects local tissue microenvironment	Small sample volume; contamination risk	Periodontal diagnosis; site-specific monitoring
Serum	Well-standardized systemic markers; high analytical reproducibility	Limited specificity to periodontal tissues	Systemic risk assessment
Saliva	Easy, non-invasive collection; repeatable sampling	Dilution effects; flow-rate variability	Broad screening; chairside testing

## Data Availability

No new data were created or analyzed in this study. Data sharing is not applicable to this article.
